# State Board of Examiners for the Degree of Doctor in Medicine

**Published:** 1864-02

**Authors:** 


### EDITORIAL DEPARTMENT.

### STATE BOARD OF EXAMINERS FOR THE DEGREE OF DOCTOR IN MEDICINE.

We are gratified to see that the initiative in this measure has been taken by the Faculty of the Buffalo Medical College, and most earnestly hope it may cause the adoption, in tangible form, of the measure, which if carried out will do more for the elevation of the profession than any other reform which could be suggested.

At the Annual Meeting of the New York State Medical Society, now being held in Albany, the following was presented and on motion adopted:

UNIVERSITY OF BUFFALO, MEDICAL DEPARTMENT.

On motion of Prof. Chas. A. Lee, seconded by Prof. James P. White, it was

RESOLVED, That the New York State Medical Society be requested to appoint a committee to consider the expediency of, and to report a plan for, the appointment of a State Board of Examiners for the degree of Doctor of Medicine, and to report at the next meeting of the Society.

RESOLVED, That the same committee be instructed to bring the subject before the next meeting of the American Medical Association, and that the delegates of this Society be instructed to urge the general adoption of the same plan in other States of the Union. Carried unanimously.

THOS. F. ROCHESTER, Chairman.

SANDFORD EASTMAN, Dean of the Faculty.

Buffalo, Feb. 2d, 1863.

This is certainly an effort in the right direction, and is exceedingly creditable to the institution suggesting it. When the graduation of students in medicine is wholly separated from the duty of teaching, and an impartial Board of Examiners shall decide who shall, and who shall not, receive the degree of Doctor in Medicine, very much will be accomplished for the elevation and advancement of the profession. It is striking at the very root of a great evil, and will meet with opposition; indeed, we have no doubt it will be overwhelmed in the almost unanimous opposition which it will meet from the various institutions now empowered to grant Diplomas. It is a measure that Medical Colleges, as such, cannot at present afford to favor, though the Professors are more fully sensible of its importance than any other individuals. There is no doubt that an impartial Board of Examiners would reject, as unprepared for the duties of the medical profession, from one-quarter to one-third of the young men who, under the present system of graduation are yearly admitted to the practice of medicine. This we believe to be true, and to be more or less applicable to all places in this country where young men are taught the primary branches of medical knowledge, and graduate, after "three years study, and two full courses of lectures, the last at this institution." The State examiners should receive compensation only from the State; should be disconnected from all schools of instruction; should, in a word, be wholly impartial.

It has been claimed that the same was accomplished by the appointment of Curators, who are invited to *attend* the examination of the students and vote upon their qualifications; and this does *appear* quite satisfactory, while in reality it accomplishes nothing. Whoever notices the manner of these appointments, the terms of this service and the opportunity afforded to determine the respective merits of individuals, will readily discover that in this way the feeblest protection is afforded the open doors of our profession, which should be guarded by ever faithful janitors.

We understand, also, that this plan of appointing State Examiners, has been considerably perfected by the Institution suggesting it, and that the details will be urged upon the consideration of the State Society at its present meeting. It appears that the Buffalo Medical College are in earnest in advocating this measure. The competition in medical schools and the desire to obtain large classes, has had prejudicial influence upon the standard of medical education, and so great has this evil become, that it it quite time for commencing a reform. Young men have been encouraged to pursue the study of medicine without preliminary preparation, and graduated without respectable professional attainment, thus lowering the professional standard, and making the degree of Doctor in Medicine a disgrace, rather than an honor. If this reform, now suggested and urged upon the profession by the Medical College in Buffalo, is favored by the other colleges in the State, we shall soon be redeemed from the power and influence of a system which has disgraced the profession, lowered its standard of attainment, reflected obloquy and contempt upon its degree, and come well nigh reducing medicine, as learned and practiced, "to the level of a trade."

It may be thought that we draw this matter in rather high colors, but the revelations of modern investigation leave no doubt that a poor doctor is vastly worse than none at all. I say revelations of modern investigation for it was formerly believed that any one who could give some medicine was useful in the absence of a physician; but it has been reserved for the physicians of our day to discover, and for the people of our times, in any measure to perceive, that medicine, *per se*, is poison, and useful only when applied under the direction of an honest, educated, intelligent physician. We sincerely hope the profession will see to it that no other than such are hereafter admitted to its ranks.

